# A comparative analysis of 2D and 3D experimental data for the identification of the parameters of computational models

**DOI:** 10.1038/s41598-023-42486-3

**Published:** 2023-09-22

**Authors:** Marilisa Cortesi, Dongli Liu, Christine Yee, Deborah J. Marsh, Caroline E. Ford

**Affiliations:** 1 Gynaecological Cancer Research Group, School of Clinical Medicine, Faculty of Medicine and Health, University of New South Wales, Kensington, NSW Australia; 2 Laboratory of Cellular and Molecular Engineering, Department of Electrical Electronic and Information Engineering “G. Marconi”, Alma Mater Studiorum-University of Bologna, Cesena, Italy; 3 Translational Oncology Group, School of Life Sciences, Faculty of Science, University of Technology Sydney, Ultimo, NSW Australia

**Keywords:** Cancer models, Biomedical engineering, Software

## Abstract

Computational models are becoming an increasingly valuable tool in biomedical research. Their accuracy and effectiveness, however, rely on the identification of suitable parameters and on appropriate validation of the in-silico framework. Both these steps are highly dependent on the experimental model used as a reference to acquire the data. Selecting the most appropriate experimental framework thus becomes key, together with the analysis of the effect of combining results from different experimental models, a common practice often necessary due to limited data availability. In this work, the same in-silico model of ovarian cancer cell growth and metastasis, was calibrated with datasets acquired from traditional 2D monolayers, 3D cell culture models or a combination of the two. The comparison between the parameters sets obtained in the different conditions, together with the corresponding simulated behaviours, is presented. It provides a framework for the study of the effect of the different experimental models on the development of computational systems. This work also provides a set of general guidelines for the comparative testing and selection of experimental models and protocols to be used for parameter optimization in computational models.

## Introduction

Computational models are becoming an increasingly important tool in biomedical research, allowing for the study of complex phenomena in controlled environments^1,2^, the prediction of a system’s behaviour in multiple conditions^3,4^, and the testing of hypotheses^5,6^. Experimental corroboration, here defined as the combination of computational model calibration and validation, is a key aspect in the development of these tools, as it represents the connection between the in-silico and the in-vitro models.

Calibrating a computational model consists of the identification of its parameters so as to recapitulate the process of interest. Multiple search and optimisation algorithms can be used in this phase^3,7–9^, although empirical parameters selection remains common when a small number of well constrained parameters needs to be identified. Validation is the procedure used to determine the accuracy of the computational model and generally involves the comparison between simulated results and experimental data not used during the calibration phase. This step is widely recognised as fundamental for the development of useful and effective computational models and a wealth of resources and guidelines are available from the recent scientific literature^4,10,11^.

The effect of the experimental model on the results of the computational model’s corroboration, however, remains largely unexplored. This is a critical aspect, as multiple evidences point to a major role of the in-vitro model in determining cell behaviour, especially when considering 3D cell cultures^12–14^. Maintaining cells in a 3D environment is becoming more and more common as their increased complexity tends to enable a more accurate replication of the behaviours observed in-vivo^15,16^. But availability of complete datasets acquired in comparable conditions can be a challenge. This often leads to the necessity of corroborating a computational model using data acquired on a combination of 2D and 3D settings. This practice has potentially deleterious effects on the accuracy and reliability of the simulated results. To test this hypothesis, we here present a comparative study of the same computational model corroborated with datasets acquired using either a 2D monolayer culture, 3D experimental models, or a combination of the two, to characterise, in a controlled and thorough manner, the effect on the computational model parameters of combining experimental data acquired from different models.

As a case study, we chose to focus on transcoelomic metastasis, the major mechanism of metastasis (or cancer spread) in ovarian cancer^17^. It occurs via the seeding of cancer cells onto the omentum, or other tissues within the abdominal cavity, following their detachment from the ovary and it is enabled by the ascites fluid which builds up in this region, and by the receptiveness of the surrounding tissues to colonisation^18–24^.

The extensive cell-cell and cell-environment interactions involved in this process have led to the development of a number of 3D cell culture models in order to research facets of this phenomenon^12,25–29^. We selected a 3D organotypic model^30,31^ used extensively to study the invasion and adhesion capabilities of ovarian cancer cells^32–36^ and 3D bio-printed multi-spheroids for the quantification of proliferation.

Together, these experimental models allow us to study both the initial phases of metastasis, when cells floating within the abdominal cavity exhibit a phenotype associated with very little proliferation, and the later stages of this process, when sustained cancer cell proliferation is observed within the omentum^37^. In all cases, standard assays performed on 2D monolayers were used as comparison.

The structure of the computational model was maintained constant throughout the analysis, to enable the comparison between the results obtained with different datasets. Changes in the parameter sets and the resulting simulations were analysed and used to draw general conclusions regarding the selection of the most appropriate experimental setting for the characterisation of each property. Validation of the computational model, through the comparison between the simulations and data not previously used during the calibration phase, provided a strategy to identify which combination of experimental data was associated with the most accurate in-silico representation of response to treatment in our model of high-grade serous ovarian cancer (HGSOC).

**Table 1:** Variables used to formalise cell behaviour and their definition.

Variable	Definition
AGE	Age of the cell normalised with respect to the length of the simulation
D	Local drug concentration normalised with respect to the amount added in the media
D_0_	Distance between the position of the current cell and the bottom of the culture (normalised between 0 and 1)
Glu	Local glucose level normalised with respect to its concentration in the media
O2	Local oxygen level normalised with respect to its concentration in the media
T_c_	Current time point normalised with respect to the length of the simulation
T_d_	Time at which the current cell died normalised with respect to the length of the simulation
TLD	Time elapsed since the last division of the current cell normalised with respect to the length of the simulation

## Methods

### Cell culture

The HGSOC cell line PEO4 was used for this study^38^. This cell line is characterised by resistance to platinum treatment and can be considered a good model of recurrent disease^39^. Cells were kindly gifted by Dr Simon Langdon (University of Edinburgh, Edinburgh, UK) and labelled with GFP (pLKO.1-Neo-computational modelV-tGFP vector from Sigma-Aldrich, USA) to enable their identification within the 3D organotypic model. Cells were maintained in RPMI medium (Thermo Fisher, Waltham, MA, USA), supplemented with 10% FBS (Sigma-Aldrich, USA), 1% Pen-strep (Sigma-Aldrich, USA) and 1% GlutaMAX (Thermo Fisher, Waltham, MA, USA).

The 3D organotypic model, chosen to evaluate adhesion and invasion, was built co-culturing PEO4 cells with healthy omentum-derived fibroblasts and mesothelial cells collected from patients undergoing surgery for benign or non-metastatic conditions patients at the Royal Hospital for Women and Prince of Wales Private Hospital (site specific approval ethics # LNR/16/POWH/236). The South Eastern Sydney Local Health District Human Research Ethics Committee (SESLHD HREC approval #16/108) approved the collection of these samples. Informed consent was obtained from all the patients participating in the study and samples were processed and analysed in accordance with relevant guidelines and regulations.

The protocol for the realization of the organotypic model is fully described in^40^. In brief, 100 $\upmu$l of a solution of media, fibroblast cells (4 $\cdot$10^4^ cells/ml) and collagen I (5 ng/$\upmu$l, Sigma-Aldrich, USA) was added to the wells of a 96-well plate. After 4 hours of incubation at 37^o^C and 5% CO_2_, 50 $\mu$l of media containing 20,000 mesothelial cells was added on top. The whole structure was maintained in standard culturing conditions for 24 h prior to seeding of cancer cells. PEO4 cells were added at a density of 1$\cdot$10^6^ cells/ml (100 $\mu$l/well) in 2% FBS media.

**Table 2:**
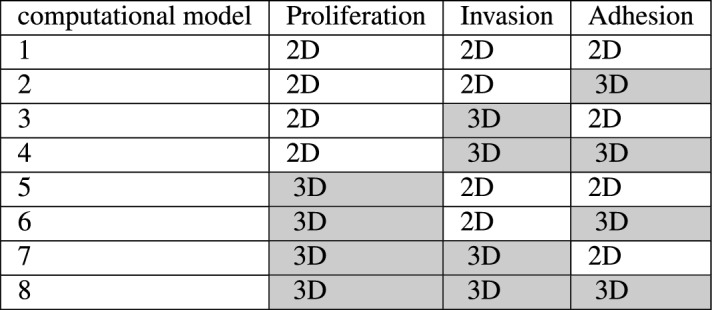
Combination of the 2D (white background) and 3D (gray background) data used for the analysis.

Proliferation was quantified in 3D multi-spheroids encapsulated in PEG-based hydrogels created using the Rastrum 3D bioprinter (Inventia Life Science, Alexandria, New South Wales)^41,42^. Three-thousands PEO4 cells per well were printed as an “Imaging model” using the Px02.31P matrix, atop an inert hydrogel base, and across an entire tissue culture-grade flat bottomed 96-well plate. The hydrogel matrix is characterised by a 1.1 kPa stiffness and by its functionalisation with arginylglycylaspartic acid (RGD), a peptide shown to promote cell adhesion^43^. Printed spheroids were maintained at 37^o^C and 5% CO_2_ for a week prior to each experiment.

### Proliferation

Proliferation in 2D was measured via MTT assay (Thermo Fisher, Waltham, MA, USA), following the manufacturer’s protocol (Supplementary Fig. 1). Briefly, PEO4 cells were seeded in 96 wells plates at a density of 10,000 cells per well. After 24 h, treatment with different concentrations of either cisplatin (50, 25, 12.5, 6.2, 3.1, 1.6, 0.8, 0.4, 0 $\mu$M) or paclitaxel (50, 25, 12.5, 6.2, 3.1, 1.6, 0.8, 0.4, 0 nM) was administered. Following 72 h of treatment a solution of 2 mg/ml of MTT was added to each well and incubated for 3 hours. The media-MTT solution was then discarded, and the formazan crystals solubilized in DMSO (Sigma-Aldrich, USA). Absorbance was measured at 570 nm. All data were normalised with respect to the untreated condition and corrected for the absorbance of RPMI medium. A total of 3 biological replicates, each comprising 3 technical replicates was analysed for condition.

Real-time monitoring of PEO4 cell growth within the hydrogel multispheroids and in the absence of treatment was conducted using an IncuCyte S3 Live Cell Analysis System (Sartorius, Gottingen, Germany). Three cell densities (2000, 3000 and 4000 cells/well in hydrogel) were considered, and the phase count function of the device’s analysis software was used to determine the number of cells, every hour over 7 days. A total of 10 wells/condition were considered for this experiment. An evaluation of viability with CellTiter-Glo 3D (Promega, Madison WI, USA) was also conducted at the end of monitoring. Real-time monitoring and the end-point assays were in agreement and a density of 3000 cells per well was chosen for further experiments with only CellTiter-Glo 3D.

Treatment with cisplatin and paclitaxel, in the same concentrations used for the 2D experiments, was administered 7 days after the printing, to allow for the establishment of a stable 3D culture. Measurements of viability using CellTiter-Glo 3D were conducted after 72 h following the manufacturer’s protocol. A total of 3 biological replicates, each comprising at least 3 technical replicates, was analysed for each condition (Supplementary Fig. 1). All data were corrected with respect to the signal produced by the matrix devoid of cells and normalised with respect to the average value measured for the untreated control.

**Table 3:** Optimal parameter configurations for each computational model in Table 2 computational models with identical parameters values (e.g. 1 and 2) will be considered as one condition (1/2) for the rest of the analysis. Each computational model has also been color-coded (last column of the table) throughout the analysis.

Computational model		Parameters					Colour
		a	b	c	d	e	
1	1/2	1	0.1	0.1	0.01	0.1	
2							
3		1	0.5	0.1	0.01	0.1	
4		1	0.5	0.1	0.1	0.1	
5	5/6	1	0.1	0.05	0.001	0.5	
6							
7	7/8	1	0.5	0.05	1	0.5	
8							

### Adhesion

Adhesion in 2D was evaluated as in^44^. Briefly the wells of a 96-well plate were coated with 10 $\upmu$g/ml of collagen I (Sigma-Aldrich, USA) or 3% BSA (Sigma-Aldrich, USA). 100,000 PEO4 cells were seeded on top of each coating and incubated for 2, 3 or 4 h at 37^o^C and 5% CO_2_. Unattached cells were then washed away, prior to fixing with 96% ethanol and staining with 1% crystal violet (Supplementary Fig. 1). Cells were then lysed with 50% acetic acid and their density was quantified with an absorbance measurement (at 595 nm). A total of 3 biological replicates each comprising 2 technical replicates was considered for this analysis. Adhesion in 3D was quantified following the same procedure, just substituting the collagen coating with the organotypic model^40^ (Supplementary Fig. 1). A limitation of this approach is that the measured absorbance integrates the signal from all the cell types present in the organotypic model. The amount of mesothelial and fibroblast cells is however assumed to be constant throughout the experiment.

**Table 4:** Optimal parameter configurations for treatment response simulation for each computational model.

Computational model	Cisplatin			Paclitaxel		
	f	g	Score	f	g	Score
1/2	0.5	0.01	1.2	0.05	0.001	1.42
3	0.5	0.5	0.16	0.005	0.5	0.05
4	1	0.005	0.26	1	0.01	0.37
5/6	0.05	0.001	0.16	0.005	0.01	0.16
7/8	1	0.01	0.05	0.01	0.005	0.05

### Invasion

Invasion in 2D was measured using Matrigel-precoated transwell chambers (Corning Life Sciences, USA). PEO4 cells were harvested with trypsin and diluted to a concentration of 1$\cdot$10^6^ cells/ml in 1% FBS media. 100 $\mu$l of the cell solution was added to each transwell insert and incubated for 48 h (Supplementary Fig. 1).

Following the incubation, transwell inserts were gently washed with PBS and fixed in 4% paraformaldehyde for 10 minutes. Wells were washed again in PBS and mounted on a microscope slide using DAPI mounting medium (Fluoroshield, Sigma-Aldrich, USA). Slides were let dry for at least 1 h prior to imaging at the microscope (Leica DM 2000 LED fitted with a Leica DFC450c camera). Ten images from different regions of the slide were acquired and the number of invaded cells counted using custom-made software further described in the next section. A total of 3 biological replicates each comprising 2 technical replicates was considered for this analysis.

**Table 5:** Percentage difference between simulated and experimental data for each computational model and experimental model.

Computational model	Cisplatin		Paclitaxel	
	2D	3D	2D	3D
1/2	59.29	4.88	21.42	7.21
3	60.89	5.61	17.84	6.78
4	24.81	2.13	7.33	1.85
5/6	71.34	6.36	24.70	8.59
7/8	51.82	4.14	18.71	4.92

Cancer cell invasion in 3D was measured in a substantially equivalent way, by substituting the Matrigel-precoated chambers with regular transwell inserts (Corning Life Ssuch, the automatic count wasciences, USA) in which the organotypic model had been seeded^40^ (Supplementary Fig. 1). Specific modifications in the counting software used for the 2D analysis allowed for the identification of the invaded cancer cells, which were producing a stable GFP signal.

### Invasion quantification software

The number of cells in each image was quantified through custom-made software written in Python (v.3.9) and freely available at https://github.com/MarilisaCortesi/cell_counter. It uses the Otsu’s method to segment the nuclei and a labelling routine to identify each segmented region and thus determine the total number of cells (Supplementary Fig. 2a,b).The accuracy of this method was evaluated by comparing the number of cells retrieved by the software for each image, with the corresponding manual count obtained by an expert user using ImageJ (Supplementary Fig. 2c). The two measures are highly correlated (R^2^ = 0.95), and their average percentage error is consistent with the inter-operator variability for this assay (about 18%^45^). As such, the automatic count was considered to be equivalent to the manual one.

For the images obtained during 3D experiments, two additional filters based on the average fluorescence intensity and area were used to separate cancer cells from fibroblasts and mesothelial cells. In particular, a cell was labelled as PEO4 if its area was between 50 and 5,000 pixels and its average fluorescence intensity was higher than that of the background (Supplementary Fig. 2d,e,f).

### SALSA modelling and computational simulations

**Figure 1:**
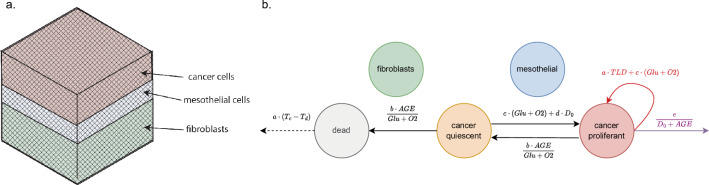
Schematic representation of the SALSA model used within this work. (**a**) Cubic lattice representing the underlying structure of the simulator. Shaded areas distinguish the three main layers of the omentum lining (fibroblast in green, mesothelial in blue and cancer in red). (**b**) Flowchart of the states (nodes) and behaviours (arcs) formalised within the computational model. Beside transitions between different states (black solid arrows) proliferating cancer cells can duplicate (red arrow) and migrate (purple arrow), while dead cells can degrade (dotted arrow). The equations on each arc represent the probability of occurrence of each rule. These are functions of several environmental and cell-specific variables. T_c_ is the current time point while T_d_ marks the time at which the current cell died. AGE is the age of the cells, while Glu and O2 represent the local concentrations of glucose and oxygen. D_0_ is the distance between the position of the cell and the bottom of the culture. These definitions are also reported in Table 1. The behaviour of fibroblasts and mesothelial cells has not been formalised. They are assumed to maintain their status and affect the behaviour of cancer cells by consuming resources (oxygen and glucose) and occupying space within the virtual tissue.

Computational simulations were conducted in SALSA^46–48^, a hybrid continuous-discrete cellular automaton freely available at https://www.mcbeng.it/en/category/software.html. The continuous component of the model solves the diffusion equation to retrieve the distribution of relevant variables (glucose, oxygen, drug concentration) throughout the simulated culture. The discrete one, on the other hand, models cell behaviour through a series of probabilistic rules describing macroscopic behaviours (e.g. migration, division, cell death). A cubic 3D lattice constitutes the main structure of the simulator (Fig. 1a). Cells can be positioned at each of the grid’s nodes and the values of the continuous variables are computed at the same locations. Specifically formatted configuration files (available as supplementary material) are used to formalize cell behaviour and initialise the experimental conditions. The simulation then proceeds for a set number of iterations, each corresponding to one hour. At each iteration the concentration of the continuous variables is updated, solving the diffusion equation and, for each cell, one of the behavioural rules is executed according to their probability. The reader is referred to^46^ or https://www.mcbeng.it/en/category/software.html for further details on the simulation structure and functioning.

Modifications to the SALSA seeding procedure were implemented to replicate the layered structure of the omentum (Fig. 1a). Fibroblasts cells were limited to the bottom half of the model, with mesothelial cells located immediately on top. Cancer cells were initially positioned above the mesothelial layer and were constrained to move toward the bottom, as the region above them represents the peritoneal cavity. The position of each cell, within the specific region, was randomly determined as in previous versions of SALSA.

Figure 1b summarises the computational representation of the 3D organotypic model. It comprises 5 different cell states: (i) fibroblasts, (ii) mesothelial cells (iii) dead PEO4 cells, (iv) quiescent PEO4 cells and (v) proliferating PEO4 cells. Arcs within the graph in Fig. 1b represent the behavioural rules and are labelled with the equations describing their probability of occurrence (see Table 1 for the definition of each variable). These rules were determined collating multiple evidence from the scientific literature, with the aim of describing the behaviour of PEO4 cells. Parameters a–e (on the arcs in Fig. 1b) are scale factors that allow to modulate the likelihood of each rule independently of the value of the variables in its probability functions. They were empirically estimated through the procedure described in the following section. Fibroblasts and mesothelial cells don’t have a formalised behaviour and are assumed to maintain their status throughout the simulation. They however consume resources (glucose and oxygen), thus impacting indirectly the behaviour of the HGSOC cells.

Treatment with cisplatin and paclitaxel was modelled as described in^47^. A sigmoid curve (S) was used to describe the probability of the drug affecting cell behaviour as a function of the local drug concentration (D). The parameters of this response curve were identified, using the IC_50_ values and assuming no effect in absence of the drug. In both cases, only proliferation (PR) and the rate of cell death (CDR) were considered to be affected by the treatment, in accordance with the mechanism of action of these agents^49,50^. Eqs. 1 and 2 show the modified probability functions for proliferating cancer cells doubling and the quiescent cancer cells death, while Supplementary Fig. 3 reports the updated state graph.

1 $$\begin{aligned} PD = a \cdot TLD + c \cdot (Glu + O2) - f \cdot S(D) \end{aligned}$$

2 $$\begin{aligned} CDR = \frac{b \cdot AGE}{Glu + O2} -g \cdot S(D) \end{aligned}$$

### Parameters estimation

Initially, the parameters describing the behaviour of the culture in absence of treatment (Fig. 1) were calibrated. A number of different values between 0 and 1 were considered for each parameter (0.001, 0.005, 0.01, 0.05, 0.1, 0.5, 1) and every possible combination of these values was simulated 3 times for the equivalent of 72 h. Regular sampling was preferred to pseudo-random methods (e.g., Latin Hypercube) as it is associated with a more thorough exploration of the parameter space when a limited number of parameters with well-defined ranges need to be identified. The replicates were then averaged and a score comparing simulated and in-vitro data was computed (Eq. 3). Supplementary Fig. 4. shows an analysis of how the score varies (Eq. 3) with the distance from the optimal configuration. For this analysis, the distance from the optimal parameter set was computed as in Eq. 4 where i is an index that varies between the parameters a and e, $p_c(i)$ is the value of the parameter i in the current configuration and $p_o(i)$ the value of the same parameter in the optimal parameter set. The median and interquartile range of the error distributions are largely conserved both across different parameter configurations and among computational models. While this might indicate that the computational models exhibit a limited range of behaviours, it also suggests that the exploration of the parameter space presented in this work is sufficient. Of note is the increase in the error range when considering the doubling rate measured in 3D (Supplementary Fig. 4e–h). While median and interquartile ranges are mostly consistent with those obtained for 2D proliferation data (Supplementary Fig. 4a–d), this might suggest an increased complexity of the behaviour measured in 3D, that fewer parameter configurations can recapitulate. Regular sampling, additionally, provides a general overview of the range of behaviours that the computational model can achieve. This characterisation was used to verify that the behavioural rules were suitable to describe transcoelomic metastasis in HGSOC. Maintaining the same possible values for all the parameters, additionally, simplifies the comparison among them and the determination of their relative importance for the recapitulation of specific behaviours.

3 $$\begin{aligned} S = S_p + S_i + S_a \end{aligned}$$

4 $$\begin{aligned} Distanc e= \sum _{i=a}^{e} |p_c(i)-p_o(i)| \end{aligned}$$

S_p_, S_i_ and S_a_ are defined as in Eqs. 5, 6 and 7, where C_x_ is the number of simulated cancer cells at T = x. EG is the expected population growth. It was set to 2 for the monolayer cultures (based on a simulation length equivalent to 72 h and a doubling time between 36 and 46 h^38^) and to 0.87 for the 3D hydrogel multispheroids. This value was obtained as the number of cells counted in bright-field images obtained during a 7 day long experiment in the IncuCyte real-time cell imaging instrument (Supplementary Fig. 5a). Finally, I_silico_ and I_vitro_ are the number of invaded HGSOC cells and A_x_ the measured amount of adherent PEO4 cells at T = x.

5 $$\begin{aligned} S_p = \frac{\frac{C_{72}}{C_0}- EG}{EG} \end{aligned}$$

6 $$\begin{aligned} S_i = \frac{I_{silico} - I_{vitro}}{I_{vitro}} \end{aligned}$$

7 $$\begin{aligned} S_a = \Sigma _{t = 2}^4 \frac{\frac{C_t}{C_4}- \frac{A_t}{A_4}}{\frac{A_t}{A_4}} \end{aligned}$$

S_p_ measures the effectiveness of the computational model in recapitulating cancer cell proliferation.

S_i_ achieves the same purpose, but it compares the number of invaded cells measured in-vitro with the average number of migration events recorded during the simulation.

S_a_ has a similar structure, but it compares the number of simulated PEO4 cells at iterations 2-4 to the corresponding results of the adhesion experiments.

These terms were assigned equal importance in the definition of the score (Eq. 3) to favour parameter configurations modelling proliferation, invasion and adhesion with the same accuracy.

The time-points at which the experimental data were acquired were selected independently for each assay, as differences in timescale and duration of the characterised processes prevented the identification of a single experiment duration.

A total of 8 different computational models were identified, using every possible combination of 2D and 3D data (Table 2) and choosing the parameter set associated with the best (i.e., lowest) score value.

The same procedure was repeated for the two parameters recapitulating the drug treatment. In this case, treatment with IC_50_ values of cisplatin and paclitaxel (10.4 $\upmu$M and 3.04 nM respectively^51^), was simulated. S_p_ (Eq. 5) was used to evaluate the performance of each configuration, with an expected population growth of 0.5, according to the definition of IC_50_.

### Computational model validation

The validation of the computational models was conducted by comparing the simulated dose response curves to cisplatin and paclitaxel, with the corresponding experimental data acquired in monolayer cultures and 3D multi-spheroids. No modification to the structure or parameters of the model was applied, with respect to the calibration stage, and the in-vitro data used for the comparison were not used to identify any of the parameters.

### Statistical analysis

The Kolmogorov–Smirnov test was used, whenever appropriate, to evaluate whether the distribution underlying the two sets of samples was the same. This method was chosen as it does not make any assumption on the shape of the underlying distribution. A p-value of 0.05 was chosen as the threshold for significance.

## Results

**Figure 2:**
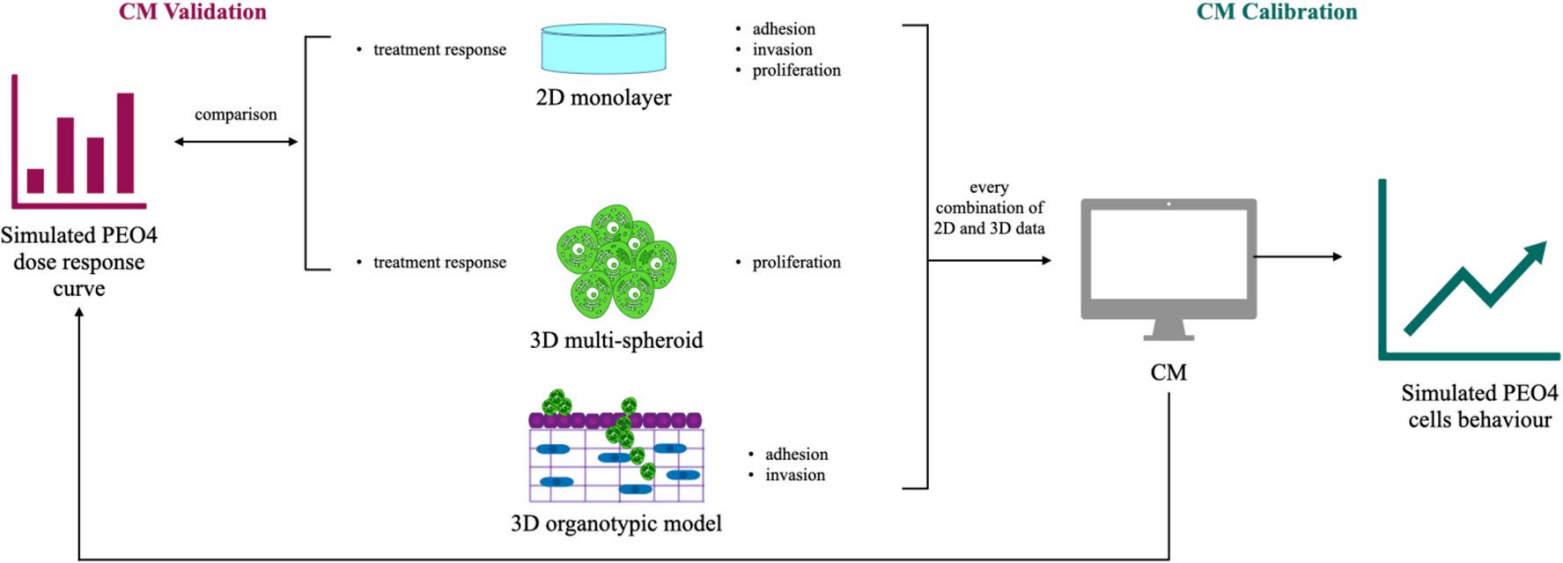
Flowchart of the analysis presented in this work. Different combinations of experimental data from 2D monolayers and 2 3D experimental models (hydrogel multi-spheroids and an organotypic model) were used to calibrate the same computational simulator of transcoelomic metastasis. These computational models were then used to simulate the response to either cisplatin or paclitaxel. The comparison between the simulated and measured dose response curves enabled the validation of the computational models and thus the determination of which computational model yields the results more closely matching experimental data.

Our analysis is schematically described in Fig. 2. We considered three experimental models: 3D hydrogel multi-spheroids, a 3D organotypic model and standard monolayer culture. The use of two 3D experimental models was required as the computational models describe both the first phases of metastasis, when cancer cells exhibit limited proliferation but readily adhere and invade and the later stages of disease progression when cells proliferate within the invaded tissue. The organotypic model is an accurate representation of the adhesion/invasion phase, but constraints on the length of the experiments and difficulties in separating the contribution of the different cell types limit its usefulness for the evaluation of proliferation. Hydrogel multi-spheroids, on the other hand, yield accurate proliferation measurements, but lack the multilayer structure useful for the study of invasion. As such, we decided to exploit the strengths of both systems and evaluate adhesion and invasion in the organotypic model and proliferation and drug response in the 3D hydrogel multi-spheroids. The same measures were also obtained in standard 2D cell cultures.

Adhesion, invasion and proliferation data were used to calibrate the computational models (right end side of Fig. 2). To test the effect of using different experimental models on the simulated results we considered all possible combinations of 2D and 3D data. The calibrated computational models allowed study of the in-silico behaviour of PEO4 cells both in absence and presence of treatment. The simulated dose response curves were compared with their experimental counterpart, acquired both in 2D monolayers and in the 3D hydrogel multi-spheroids, to determine which combination of calibration data yielded the computational model better replicating the treatment response of PEO4 cells (left end side of Fig. 2).

### In-vitro quantification of adhesion and invasion

**Figure 3:**
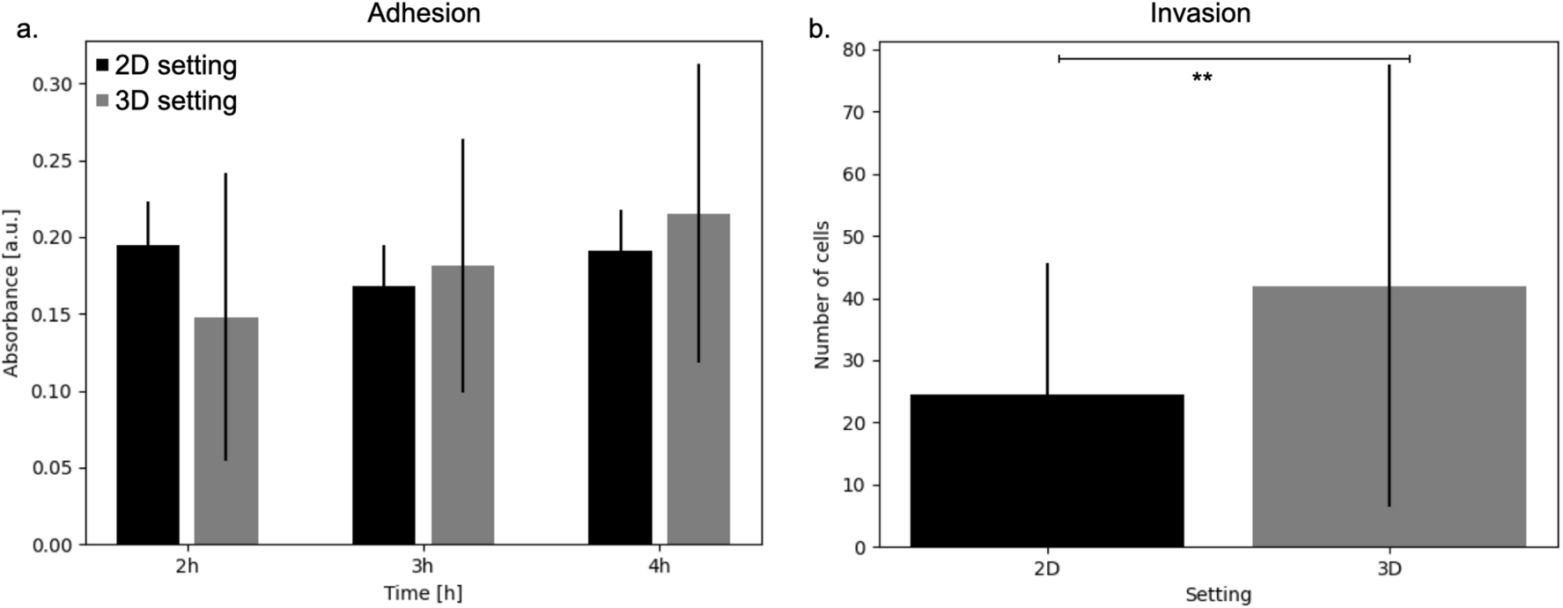
Experimentally measured adhesion and invasion in both 2D and 3D experimental models. (**a**) Adhesion measurements at 2, 3 and 4 h post seeding. In 2D a collagen coating was used as substrate while in 3D HGSOC cells were seeded on the organotypic model. (**b**) Average number of invaded PEO4 cells in 2D and 3D (Kolmogorov Smirnov p value = 0.005). In both panels error bars represent the standard deviation (n = 3).

Figure 3a reports the results of the adhesion time course conducted in both 2D monolayers and the organotypic model. Very similar absorbance values were obtained for the two conditions, even though a 3D setting was associated with increased variability. Additionally, the number of adherent cells remained approximately constant within the considered timeframe, even though a slight trend appears to be present for the 3D setting.

Changing the experimental model had a more pronounced effect when quantifying invasion (Fig. 3b). Here the use of the organotypic model resulted in a noticeable increase in the number of invading cells (Kolmogorov–Smirnov test p = 0.005).

These results, together with the doubling time for PEO4 cells^38^ and their confluency measurement obtained with the IncuCyte (Supplementary Fig. 5), were used to calibrate the computational models, that is to identify which parameter sets better approximate the different combination of experimental data.

### Computational models calibration

To determine the role of the experimental model in the identification of the computational model parameters we calibrated eight different computational models, each corresponding to a different combination of experimental data (Table 2). As described in the methods section, a score was computed for each simulated parameters configuration (Eq. 3), and the one associated with the lowest value was selected.

**Figure 4:**
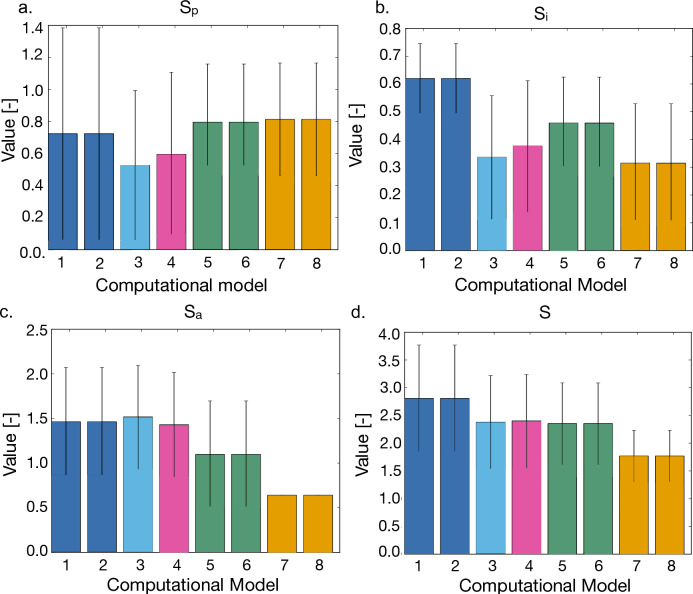
Score values associated with the best parameter configuration for each computational model Panels (**a**–**c**) refer to one of the three score components (a. S_p_, b. S_i_, c. S_a_), while panel d. shows each configuration overall score. As-per their definition (see Eqs. 3, 5, 6, 7) low score values are associated with higher accuracy of the computational model. Scores were computed independently for each simulation and then averaged (error bars represent the standard deviation). The experimental data used to calibrate each computational model are summarised in Table 2 and the corresponding colour coding is reported in Table 3.

Figure 4 reports the results of this analysis both as individual score components (panels a. to c.) and overall score value (panel d.). For each computational model the score values are reported as average and standard deviation (computed over 50 simulations). No statistical analysis was conducted on these data, as each computational model was compared to its own reference dataset. This change in experimental data, together with other factors (e.g., a different variability in the simulated data) could affect the shape of the cumulative distributions of the score and thus have a non-negligible effect on the results of the Kolmogorov–Smirnov test. Comparing the distribution averages, while limited to the available samples, is more robust to these changes and as such it was preferred.

The use of experimental data acquired in 3D seems to be associated with an overall lower score (1.7 for computational model 8 vs 2.8 for computational model 1), but the main result of this analysis is the low relevance of the experimental model used to evaluate adhesion. Indeed, in most cases, both 2D and 3D experimental data yield the same parameter set (Table 3). The only exceptions are computational models 3 and 4, which are however associated with very similar configurations (Table 3) and overall score (Fig. 4).

The parameters describing drug response were determined in the same way. Table 4 reports the values of f and g (Eqs. 1 and 2) for each computational model and the corresponding score value. Again, the error associated with the use of 3D data is generally lower, even though no general association between use of specific experimental models and parameter values was observed.

### Computational model validation

**Figure 5:**
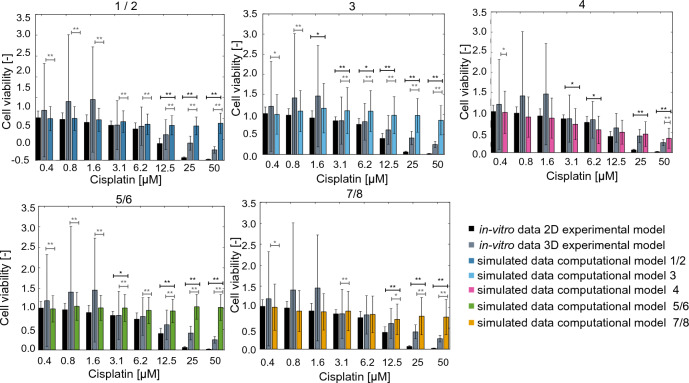
Comparison between the simulated cisplatin response (colour coded for each model as in Table 3) and the experimental data acquired in 2D (black bars) and 3D (gray bars) experimental models. In all cases, the cell viability is normalised with respect to untreated condition and data are reported as mean +/− standard deviation (n = 3 for the experimental data, n = 50 for the simulated results) Statistical testing conducted using the Kolmogorov–Smirnov test and reported in black when comparing simulated and 2D data and in grey for simulated and 3D data, * p<0.05, **p<0.01.

**Figure 6:**
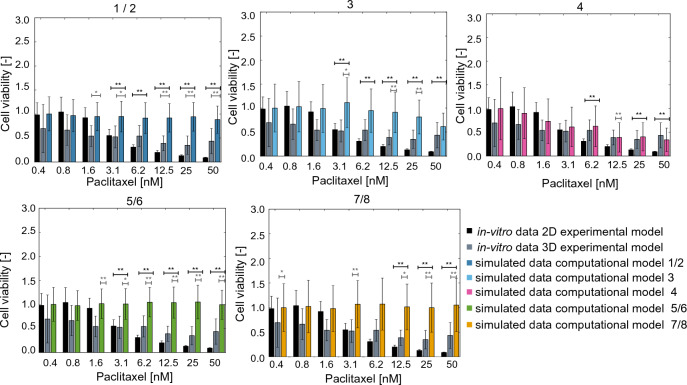
Comparison between the simulated paclitaxel response (colour coded for each model as in Table 3) and the experimental data acquired in 2D (black bars) and 3D (gray bars) experimental models. In all cases, the cell viability is normalised with respect to untreated condition and data are reported as mean +/− standard deviation (n = 3 for the experimental data, n = 50 for the simulated results) Statistical testing conducted using the Kolmogorov–Smirnov test and reported in black when comparing simulated and 2D data and in grey for simulated and 3D data, *p<0.05, **p<0.01.

Following the identification of the computational models, we compared the simulated response to treatment with either cisplatin (Fig. 5) or paclitaxel (Fig. 6) to the experimentally measured values in a 2D or 3D setting. In particular, each computational model (bars color-coded as in Table 3) was independently analysed for its ability to recapitulate the experimental dose response curves (black and grey bars for 2D and 3D data respectively). Most of the computational models showed limited response to treatment and a statistically significant difference between the simulated and experimental data (Kolmogorov–Smirnov test, * p < 0.05, ** p<0.01), especially at the higher drug concentrations. The Kolmogorov–Smirnov test compares cumulative distributions and, as such, could be affected by a change in standard deviation between simulated and experimental data. Despite this drawback, we can conclude that computational model 4 recapitulates the dose response curve accurately.

It corresponds to using 3D data for both invasion and adhesion measurements and 2D measurements for proliferation (Table 2). This result is also confirmed by relative error between experimental and simulated data (Table 5). In this case the relative change between experimental (E([D])) and simulated (S([D])) results was computed (Eq. 8) for each drug concentration ([D]), and then summed to provide an indication of how well the model captures the entire dose response curve.

8 $$\begin{aligned} Error([D]) = \frac{|S([D])- E([D])|}{E([D])} \end{aligned}$$

3D data tend to be better approximated by the computational model, as the error is lower. This might be partly due to the more limited growth rate and response to treatment observed in 3D, which might be simpler to capture with the simulator. At the same time, this difference between treatment in 2D and 3D settings is commonly observed, both in terms of increased IC_50_ and reduced overall response^52,53^.

### High resolution analysis of simulated cell behaviour

**Figure 7:**
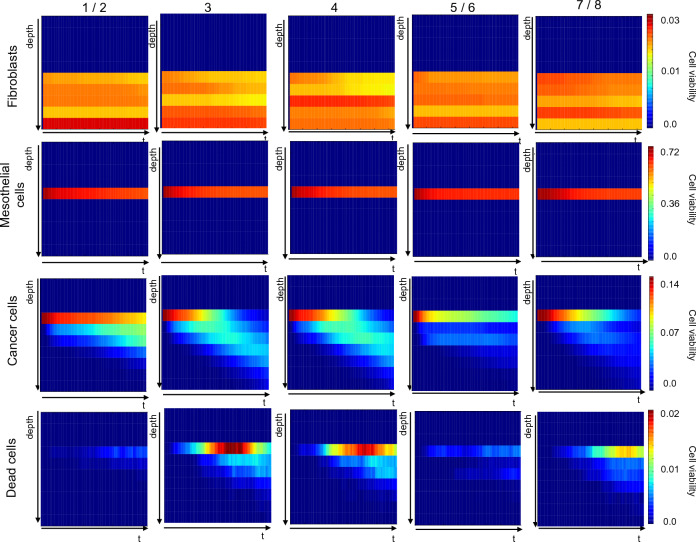
Analysis of the dynamic behaviour of simulated cell types with sub-organotypic model resolution. Each column corresponds to a different computational model, while each row is associated to a cell type. Every panel shows the average density of that cell type (over 50 simulations) over time and as a function of the z coordinate. Colour shading in each block represents cell viability (refer to scale on right end side), with dark blue and bright red representing the lower and higher cell densities. All values have been normalised with respect to total initial cell number. Heatmaps generated using a custom script in Python 3.9 and matplotlib 3.5.1).

**Figure 8:**
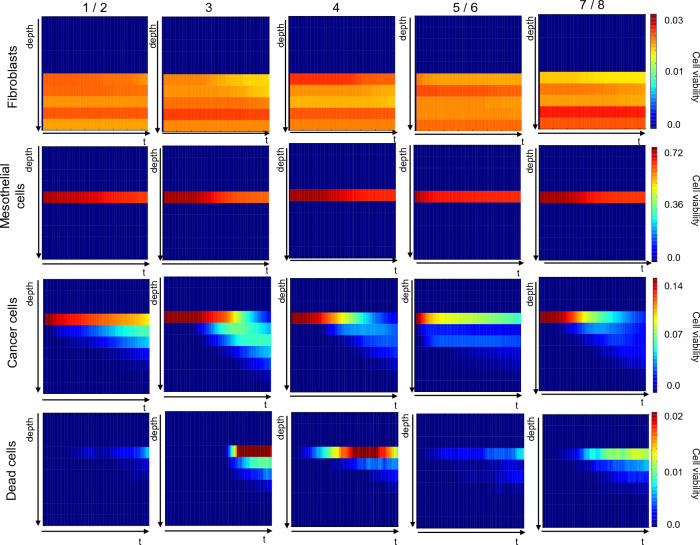
Analysis of the dynamic behaviour of simulated cell types with sub-organotypic model resolution following treatment with cisplatin at IC$_{50}$ levels. Each column corresponds to a different computational model, while each row is associated to a cell type. Every panel shows the average density of that cell type (over 50 simulations) over time and as a function of the z coordinate. Colour shading in each block represents cell viability (refer to scale on right end side), with dark blue and bright red representing the lower and higher cell densities. All values have been normalised with respect to total initial cell number. Heatmaps generated using a custom script in Python 3.9 and matplotlib 3.5.1).

**Figure 9:**
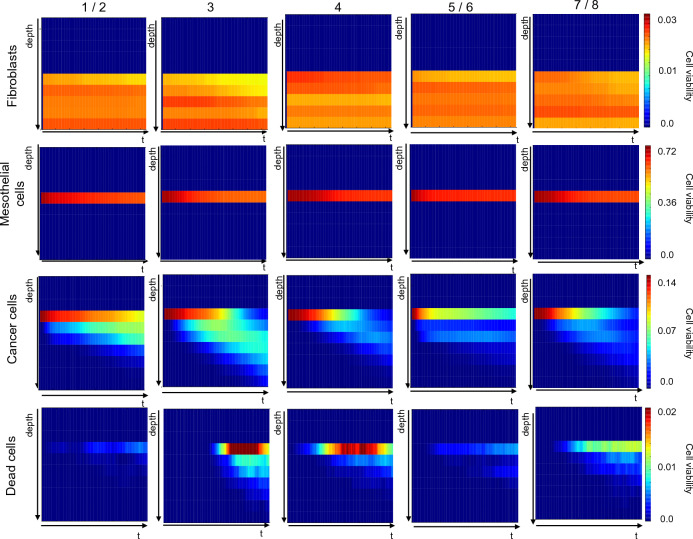
Analysis of the dynamic behaviour of simulated cell types with sub-organotypic model resolution following treatment with paclitaxel at IC$_{50}$ levels. Each column corresponds to a different computational model, while each row is associated to a cell type. Every panel shows the average density of that cell type (over 50 simulations) over time and as a function of the z coordinate. Colour shading in each block represents cell viability (refer to scale on right end side), with dark blue and bright red representing the lower and higher cell densities. All values have been normalised with respect to total initial cell number. Heatmaps generated using a custom script in Python 3.9 and matplotlib 3.5.1).

A key feature of SALSA is that it retains information on the position of each cell at each iteration. This enables study of the dynamic distribution of each population with sub-organoid resolution. In particular, the distribution of the average cell viability for each simulated cell type was computed as a function of time (x axis) and z coordinate (y axis). Cell viability was obtained, for each simulation, normalising the number of cells at each depth by the initial population cardinality. Averaging over the simulation yielded the heatmaps in Figs. 7, 8 and 9.

The difference between them is the treatment condition: Fig. 7 outlines the behaviour of the different computational models in the absence of treatment while Figs. 8 and 9 refer to treatment with cisplatin and paclitaxel at a concentration equal to the simulated IC_50_. Fibroblast and mesothelial cells are concentrated in very specific regions of the virtual organoid, in accordance with the definition of the experimental model^30,31^ and their density is constant throughout the experiment, as per computational model definition (Fig. 1). Any variation in the colour shade is due to the fact that virtual cells are randomly assigned a position within the allowed area, and this will result in a non-uniform distribution even when the average density is computed. Additionally, as virtual PEO4 cells move through the organoid they have the power of displacing fibroblasts and mesothelial cells, hence producing the slight decrease in the density of these cells observed as time progresses.

The behaviour of virtual PEO4 cells is conceptually the same in all models: their initial location is on top of the mesothelial cells, and they progressively infiltrate the underlying layers. The different computational models are however characterised by varying degrees of infiltration and proliferation within the organoid.

Configurations calibrated with the 3D invasion data (computational models 3, 4, 7/8) result in a more extensive infiltration (i.e., cancer cells present at lower depth values) while 2D proliferation (computational models 1/2, 3 and 4) is associated with an overall higher number of cancer cells. 3D invasion is also connected with a higher rate of PEO4 cell death especially in the shallower layers of the organoid and the second half of the simulation. This might be connected to the dependence of cell death on the age of the cancer cells, which is likely to be higher toward the end of the simulation and in the region where this type of cells was initially located.

Comparing Fig. 7 with Figs. 8 and 9 highlights the effect of the treatments on the behaviours of the different cell types.

Simulated fibroblasts and mesothelial cells are not affected by the treatment, as their interaction with cisplatin and paclitaxel has not been formalised. PEO4 cells, on the other hand, have a generally reduced growth and more limited infiltration within the virtual organotypic model. This seems to be connected to a delay in both these processes, whose probability increases as the effective drug concentration decreases due to degradation.

Cell death is also increased in the presence of treatment and this process seems to be more sustained over time. Surprisingly however, the number of dead cells becomes relevant at about the same time as in the untreated condition, suggesting that, in our simulations, treatment alone is not sufficient to induce cell quiescence and death. This is consistent with the treatment resistant nature of PEO4 cells but might also reflect the need for a more detailed modelling of drug response.

## Discussion

Computational modelling has acquired great relevance in biomedical and cancer research, both as a tool for fundamental research^54–56^ and as an aid for clinical decision making^57–59^. As such, an in-depth analysis of the calibration and validation procedures is necessary to maximise the utility and accuracy of these models.

The focus of this work has been the effect of using different experimental models to calibrate and/or validate the computational simulator and how this choice affects the in-silico results. To this end, we measured ovarian cancer cell proliferation, adhesion and invasion in both 2D monolayers and 3D cultures and evaluated the consequences of using distinct combinations of these data for the corroboration of the same computational system, a virtual representation of transcoelomic metastasis realised in SALSA (Fig. 1). This biological process was chosen as it is highly dependent on the 3D interaction between different kinds of the cells and their environment, a feature expected to magnify the difference between more accurate experimental models and simplified systems.

The use of different datasets led to the identification of computational models characterised by distinct parameters (Tables 3, 4) and a varying degree of accuracy when compared with the experimental data (Fig. 4, Table 4). From these results, the use of at least some of data acquired in a 3D setting tends to be associated with a lower error, underscoring the importance of accurate in-vitro models for the corroboration of in-silico systems.

It is also worth noticing the dependence of some rates on specific experimental data. The value of the b parameter, which modulates the death rate and the transition between proliferative and quiescent states (Table 3), is strictly associated with the experimental model used to evaluate invasion, suggesting that culture in the organotypic model increases the likelihood of both these phenomena. On the other hand, the values of c and e (Table 3) are mainly determined by the setting used to measure proliferation. As such, 2D monolayer seems to favour proliferation, while growing in 3D promotes migration and invasion. This is qualitatively coherent with with evidence from the literature^37^ and is also confirmed by our experimental data, as the average number of cells able to invade through the organotypic model is almost double that measured in the 2D setting (Fig. 3b). These considerations are also supported by the results presented in Fig. 7, where simulated dynamic evolution of the density of the different cell types is presented as a function of their position within the organotypic model. computational models calibrated with 3D invasion data (computational models 3, 4, 7 / 8) are associated with a deeper infiltration of cancer cells and a higher density of dead cells. 3D proliferation (computational models 5/6 and 7/8), on the other hand results in an overall lower number of cancer cells, in agreement with the in-vitro data used for the calibration.

The experimental model used to evaluate cell adhesion seems to be less relevant with 6 out of 8 models being invariant with respect to this property. This could be due to the similarity between the measurements obtained in the two experimental setups (Fig. 3a) but is also a reflection of the mainly surface nature of this phenomenon, which might resent less from the simplification in the experimental setting.

Another key difference between the experimental data measured in 2D and 3D is the increase in variability in the latter (Figs. 3, 5, 6). This is a phenomenon frequently observed when transitioning from monolayer cultures to more complex setups which has been linked to differences in the microenvironments experienced by each cell and have been shown to improve resilience and adaptability^60^.

The validation of these computational models was conducted by comparing the simulated dose response curves to cisplatin and paclitaxel with the corresponding experimental results obtained in 2D monolayers and 3D hydrogel models (Figs. 5, 6). Data acquired in the multi-spheroid model are associated with a reduced response to the treatments (gray vs black bars in Figs. 5, 6) and with an increase in variability. Most computational models are also associated with a limited response to treatment and a low sensitivity to the change in drug concentration. This is likely dependent on the formalization of cancer cell behaviour and response to treatment used within this work, and further analysis on how changing the computational model structure and probabilities affects the simulated results is warranted. Beside potentially improving the simulated drug response, this perspective study would also shed light on the role of each simulated variable (e.g., nutrients availability, cell age) in determining cell behaviour, and could provide useful insights on the biology of HGSOC cells. One of the simulated computational models (computational model 4), however, was able to effectively recapitulate 2D and 3D dose response curves. It was calibrated using 2D proliferation data and 3D invasion and adhesion measurements. Furthermore, the drug response parameters for this configuration feature a high rate of proliferation inhibition and a comparatively low induction of cell death. Overall, these characteristics produce a response comparable to model 8, which was calibrated using only 3D data, at low and mid drug levels (Figs. 5, 6, 8, 9). It however produces a better response when higher concentrations of treatment are simulated. Particularly relevant is the comparison with computational model 3, which exhibited a substantially equivalent behaviour in absence of treatment (Fig. 4) but a much more drug-resistant phenotype (Figs. 5, 6). The values of parameters f and g for these configurations suggest that, at least in this configuration, inhibiting cell proliferation might be a more effective treatment strategy than inducing cell death. Of note is also the change score rank between calibration and validation (Fig. 4, Table 4). Differences in the experimental data used as reference, and the addition of treatment response in the latter computational model are likely responsible for this variation.

Overall, while the results of this work might not be directly transferrable to different experimental and computational models, this analysis allows to draw three conclusions with general applicability.

Firstly, the interaction of multiple phenomena can result in different datasets producing similar results. This is a longstanding problem in stochastic computational modelling^61–63^, known as model identifiability, which can limit the predictive power of the computational model. An example of this phenomenon can be observed in Figs. 8, 9, where computational models 4 and 7 / 8 yield comparable cancer cells densities, despite having been calibrated with different datasets. In this case, a higher number of dead cells in computational model 4 compensates for the faster proliferation of this configuration. The comparative analysis presented in this work can be a useful tool for the identification of these compensatory mechanisms, thus enabling a deeper understanding of the computational models and their mechanisms.

Secondly, maximising the similarity between the simulated and experimental setups is associated with a low error throughout all the analysis. Indeed, while a combination of 2D and 3D data most accurately captured the response to cisplatin and paclitaxel, exclusively using data measured in 3D settings resulted in the best score for the calibration stage (Fig. 3 and second best in the drug response (Tables 4, 5).

Thirdly, behaviours more strictly connected with the interaction between the cells and the 3D environment seem to be affected more deeply by simplifications of the experimental model. In our experiments, the number of invaded cells almost doubled shifting from a 2D to a 3D setting (Fig. 3b) and the behaviour of models corroborated with 3D invasion data was markedly different from their 2D counterpart. On the other hand, quantifying adhesion in a 2D or 3D setting had little effect on the computational model’s results. As such, should it not be possible to use exclusively 3D experimental models, properties characterised by limited cell-cell and cell-environment interactions are expected to be the less affected by the simplification of the experimental model.

Overall, many obstacles are still in the way of a complete integration of in-silico and in-vitro analyses, but this and other works provide important insights on how these issues can be addressed and workflows adapted to reap the benefits of computational analysis for the study of complex biological processes.

### Supplementary Information

Supplementary Information.

## Data Availability

The experimental and simulated data have been uploaded to Zenodo (doi: 10.5281/zenodo.7939591). SALSA can be downloaded at https://www.mcbeng.it/en/ while the image analysis software for the analysis of the invasion assay is available at https://github.com/MarilisaCortesi/cell_counter. This work has also been uploaded to BioRxiv (https://doi.org/10.1101/2023.05.17.541071).
