# Author Correction: A comparative analysis of 2D and 3D experimental data for the identification of the parameters of computational models

**DOI:** 10.1038/s41598-023-50403-x

**Published:** 2024-01-09

**Authors:** Marilisa Cortesi, Dongli Liu, Christine Yee, Deborah J. Marsh, Caroline E. Ford

**Affiliations:** 1 Gynaecological Cancer Research Group, School of Clinical Medicine, Faculty of Medicine and Health, University of New South Wales, Kensington, NSW Australia; 2 Laboratory of Cellular and Molecular Engineering, Department of Electrical Electronic and Information Engineering “G. Marconi”, Alma Mater Studiorum-University of Bologna, Cesena, Italy; 3 Translational Oncology Group, School of Life Sciences, Faculty of Science, University of Technology Sydney, Ultimo, NSW Australia

Correction to: *Scientific Reports* 10.1038/s41598-023-42486-3, published online 22 September 2023

The Funding section in the original version of this Article was omitted. The Funding section now reads:

“This project has received funding from the European Union’s Horizon 2020 research and innovation programme under the Marie Sklodowska-Curie grant agreement No 883172.”

The original Article has been corrected.

