# Supplementary material for: A comparative analysis of 2D and 3D experimental data for the identification of the parameters of computational models
Source: Sci Rep. 2023 Sep 22;13:15769. doi: 10.1038/s41598-023-42486-3 (PMC10517149; doi:10.1038/s41598-023-42486-3)
Supplement: Supplementary file 1 — Supplementary Information. [file 41598_2023_42486_MOESM1_ESM.pdf]

Supplementary Material

1. Supplementary Data

1.1. SALSA configuration file

In the following the configuration file used to describe the organotypic model in SALSA is reported.

```
MODEL:

 Cell Types:

 Cell Line = organoid
 1 = Dead
 2= Cancer_Quiescent
 3 = Cancer_Replication
 4 = Fibroblasts
5 = Mesothelial

Rules:

0 = 1->0, a*(TIME-TD)
1 = 1->1, 1-(a*(TIME-TD))
2 = 1->0, environment (Glc)
3 = 1->0, environment (O2)
4 = 2-> 1, b*(AGE)/(Glc+O2) - f*DRUG1
5 = 2-> 3, c*(Glc+O2) + d*D0
6 = 2-> 2, 1-(b*(AGE)/(Glc+O2)-f*DRUG1) - (c*(Glc+O2)+d*D0)
7 = 2->0.5*U, environment (Glc)
8 = 2->0.5*U, environment (O2)
9 = 3->2, b*(AGE)/(Glc+O2)
10 = 3->3+3, a*TLD+ c*(O2+Glc) -g*DRUG1
11 = 3->0+3, e/(D0+AGE)
12 = 3->3, 1-(b*(AGE)/(Glc+O2))-(a*TLD+c*(Glc+O2)-g*DRUG1)-(e/(D0+AGE))
13 = 3->U, environment (Glc)
14 = 3->U, environment (O2)
15 = 4 ->4, 1
16 = 4->0.5*U, environment (Glc)
17 = 4->0.5*U, environment (O2)
18 = 5 ->5, 1
19 = 5->0.5*U, environment (Glc)
20 = 5->0.5*U, environment (O2)

Scaffold:

material = organoid
side [cm] = 0.6
layers = 10
porosity [%] = 87

INITIAL CONDITIONS:
```

Cell Types:

1 = 0
2 = 14
3 = 0
4 = 14
5 = 71
total=28K

Scaffold:

seeding = Organoid
iterations = 72
media = RPMI
media replace frequency [day]= 3
volume per scaffold [ml] = 0.2
flow rate [ml/h] = 0

Treatment:

ID = 1
name = **drug_name**
dose [ug/ml] = **drug_dose**
treatment = [0]
type = media

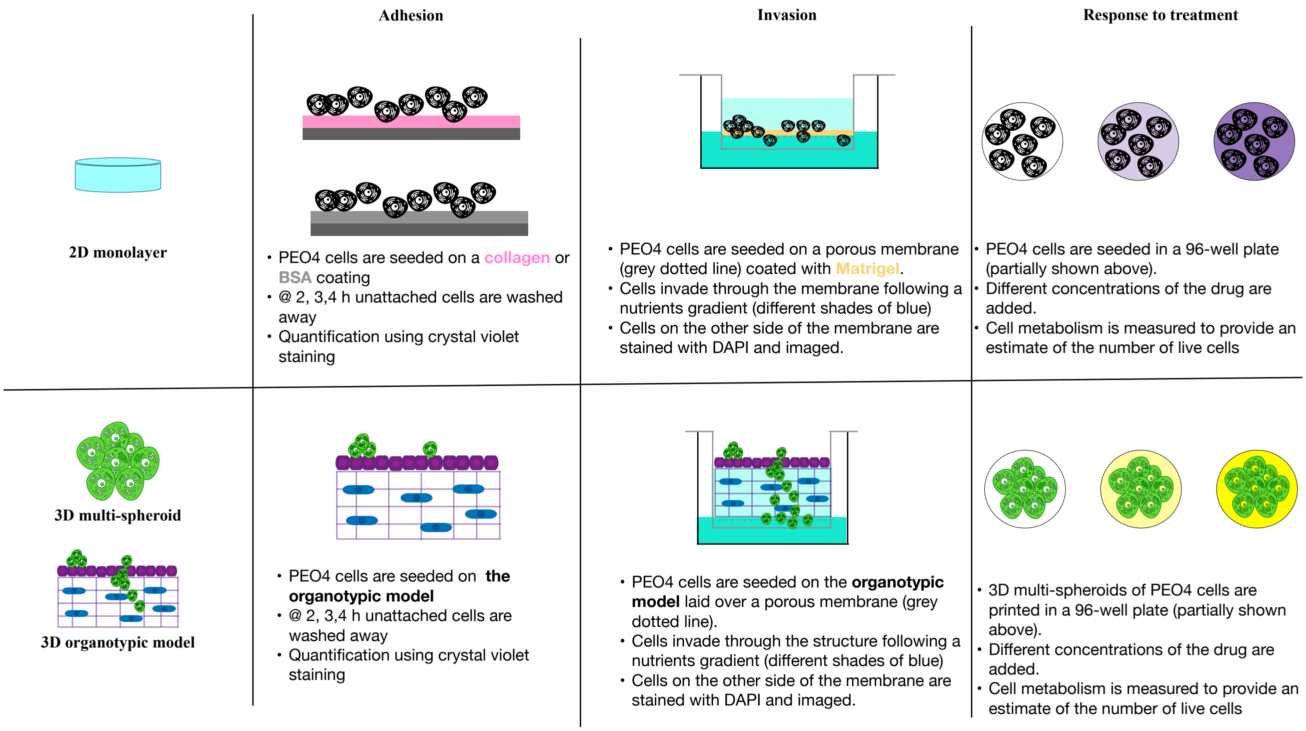

Supplementary Figure 1: Summary of the experimental analysis conducted in this work. For each considered experimental model (2D monolayer, 3D multi-spheroid and 3D organotypic model a schematic representation of adhesion, invasion and response to treatment assays is shown together with a brief description of the main steps of these protocols

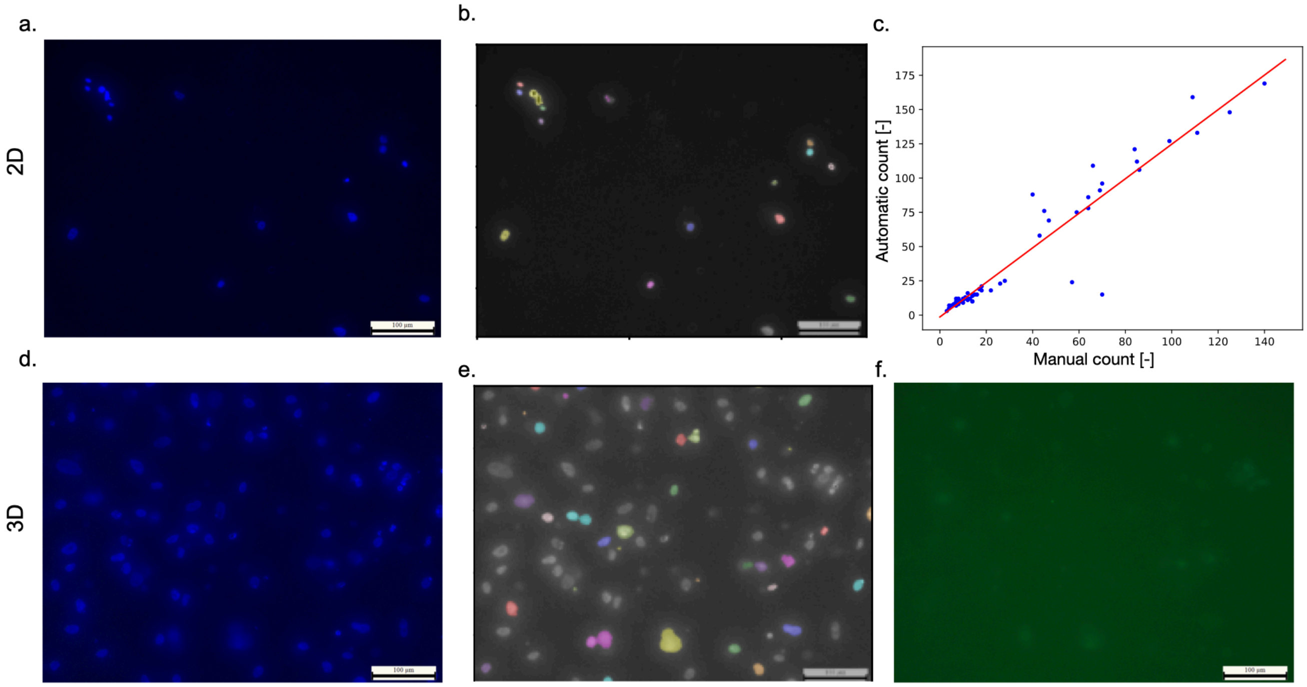

Supplementary Figure 2: Software for the analysis of the images from transwell invasion assays. a. Representative image from the 2D dataset. The cells' nuclei are stained with DAPI. b. Segmentation of the image in a. Colour overlays identify individual cells. c. Comparison between the manual and automatic counts obtained on the same images ($R^2 = 0.95$). All the images in the 2 dataset were used for this analysis. d. Representative image of the stained nuclei for the 3D dataset. e. Segmentation of the image in d. In this case only cells recognised as cancer were overlayed with different colors. f. GFP channel for the image in d. Only cancer cells express this fluorescent protein.

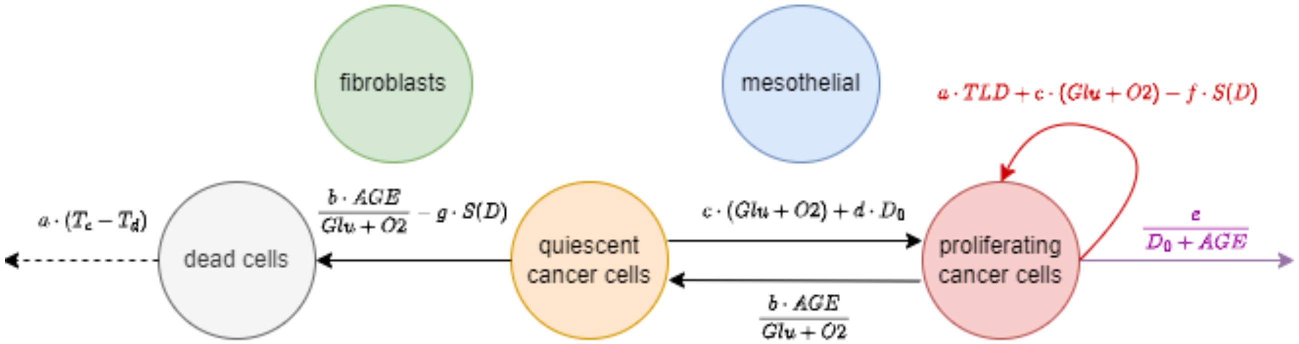

Supplementary Figure 3: Diagram outlining the behaviour of the simulated cells upon introduction of drug treatment. This is a modified version of Figure 1b, in which the proliferation probability and the rate of cell death have been modified to include the effect of cisplatin/paclitaxel. The definition of the terms and variables in this figure are in the label of Fig.1., Tab. 1 and the methods section.

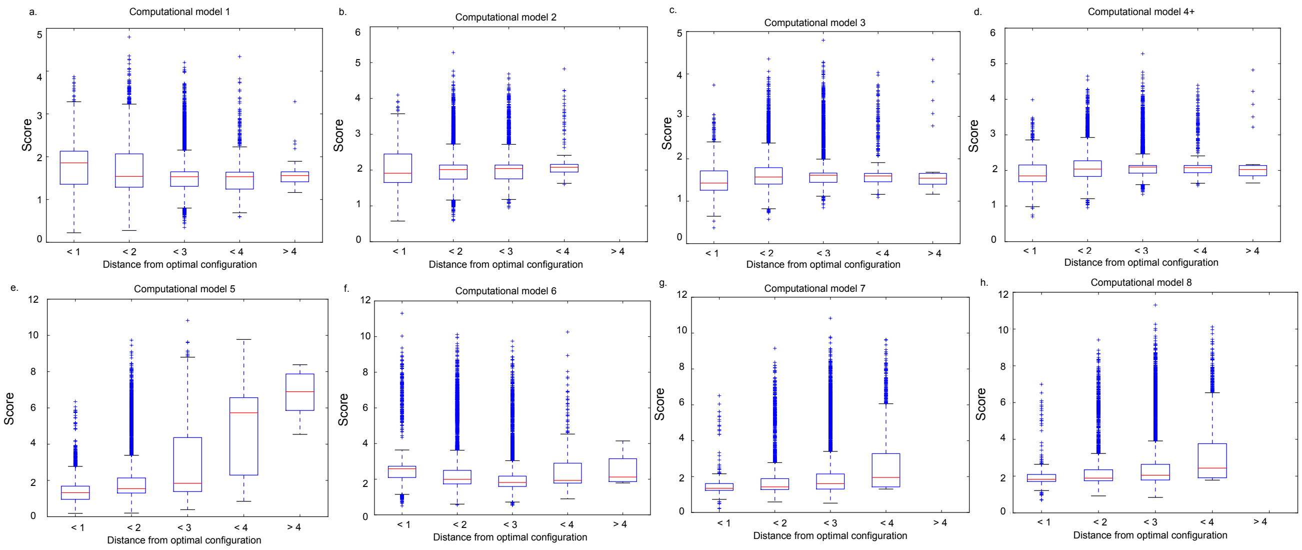

Supplementary Figure 4: Representation of the score distribution for each computational model as a function of the distance from the optimal configuration. The experimental data used for the calibration of each model are summarised in Tab 2, while Tab. 3 shows the parameters for each model. The score was computed as shown in Eq. 3 while the formula for the distance from the optimal configuration is in Eq. 4

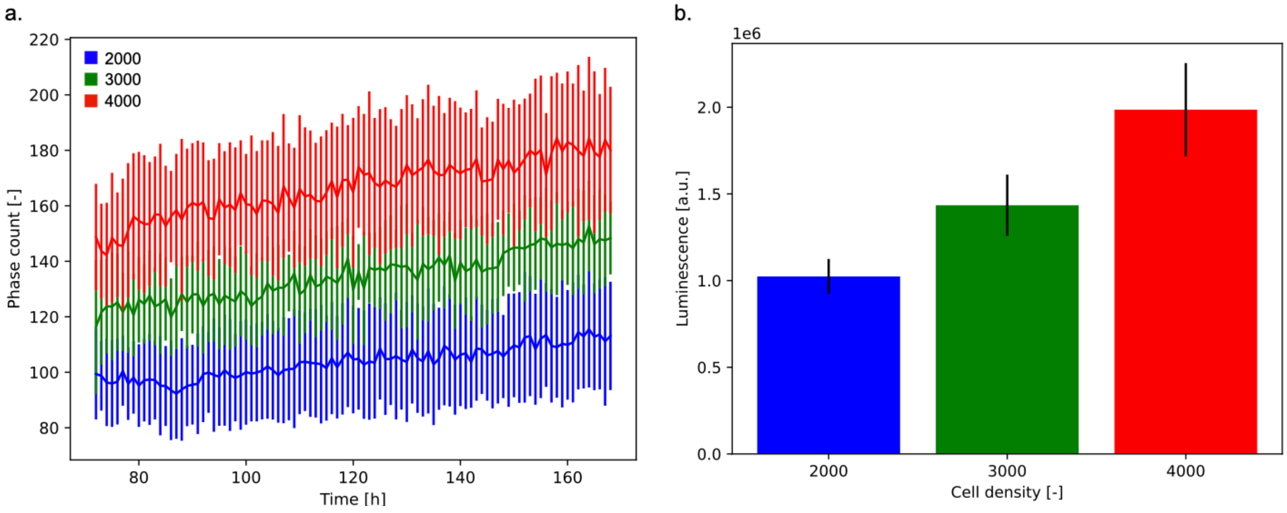

Supplementary Figure 5: Analysis of cell growth in the 3D multispheroid model. a. Tracking of the number of cells (phase count) obtained from images stacks acquired using an Incucyte S3 Live Cell Analysis System over a period of 7 days. b. End point measurements with the CellTiter-Glo 3D kit. The results obtained with the latter are equivalent to the endpoint measurements in the latter.
